# Modification of hempseed protein isolate using a novel two-stage method applying high-pressure homogenization coupled with high-intensity ultrasound

**DOI:** 10.1016/j.ultsonch.2024.107177

**Published:** 2024-11-26

**Authors:** Ruyu Zhang, Wangang Zhang, Xuan Dong, Meng Wai Woo, Siew Young Quek

**Affiliations:** a State Key Laboratory of Meat Quality Control and Cultured Meat Development, Key Laboratory of Meat Products Processing, Ministry of Agriculture, Jiangsu Collaborative Innovation Center of Meat Production and Processing, Quality and Safety Control, College of Food Science and Technology, Nanjing Agricultural University, Nanjing 210095, China; b Food Science, School of Chemical Sciences, The University of Auckland, Auckland 1010, New Zealand; c School of Biological and Food Engineering, Chuzhou University, Chuzhou 239000, China; d Riddet Institute, Centre of Research Excellence in Food Research, Palmerston North 4474, New Zealand; e Department of Chemical and Materials Engineering, Faculty of Engineering, The University of Auckland, Auckland 1010, New Zealand

**Keywords:** Plant protein, Protein modification, Physicochemical properties, Protein functionality, Structure-functional relationship

## Abstract

Hempseed protein isolate (HPI), a novel plant protein, possesses advantages as an alternative food protein from a nutritional and sustainable perspective. This study investigated HPI modification by examining the effects of high-pressure homogenization combined with high-intensity ultrasound (HPH + HIU) on the physicochemical and functionality of HPI. Firstly, the optimal homogenization pressure (180 MPa) was selected based on the solubility and particle size of HPI. Then, the effect of ultrasonic treatment time (2, 5, and 10 min) was studied at the optimal homogenization pressure. The results showed increased solubility of HPI after all treatments. Particularly, the HPH + HIU_2min_ treatment had a synergistic effect that maximumly increased the solubility of HPI from 6.88 % to 22.89 % at neutral pH. This treatment significantly decreased the HPI’s particle size, β-sheet and total sulfhydryl contents while maximizing the random coil level, intrinsic fluorescence intensity and surface hydrophobicity compared to the single HPH or HIU_2min_ treatments. The protein structure was modified and unfolded, enhancing the water-protein and oil-protein interactions, as reflected in the increase in water and oil absorption, foaming and emulsifying properties. However, extending the ultrasonic time to 5 min for the HPH + HIU treatment increased protein particle size and weakened the functional properties of HPI. Further prolonging the ultrasonic time to 10 min partially loosened the protein aggregates and restored the functional properties of HPI to some extent. The findings indicate a promising application of HPH + HIU as an efficient way for HPI modification to facilitate its broader application in the food industry.

## Highlights

• Combination of high-pressure homogenization (HPH) and ultrasound altered the HPI.
• The HPH coupled with 2 min of ultrasound maximally improved the solubility of HPI.
• HPI after HPH coupled with 2 min of ultrasound treatment had a loose structure.
• The structural change allowed HPI to effectively improve its functional properties.
• Extending ultrasonic time in coupled treatment deteriorated HPI’s functionality.

## Graphical abstract

**Figure:**
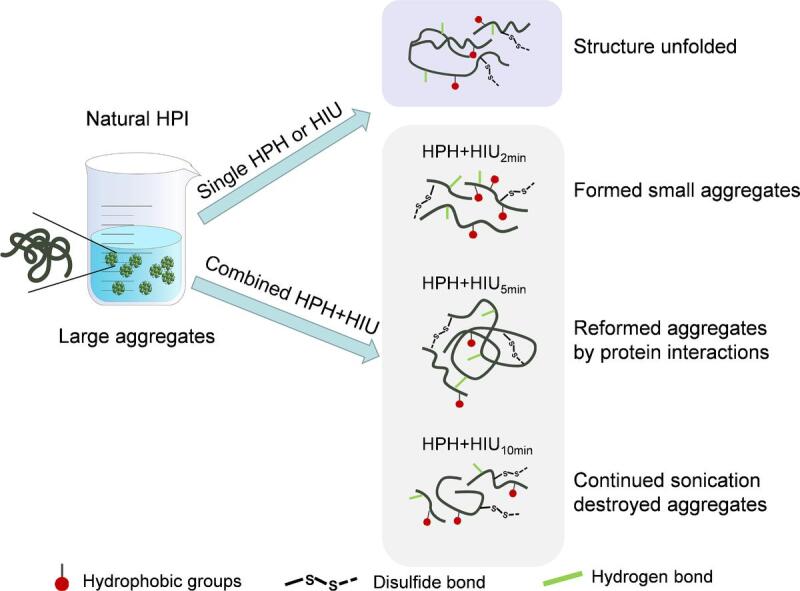

## 1 Introduction

The global population growth has increased the demand for food protein [1]. Compared to traditional animal-based proteins, plant-based proteins have advantages in sustainability, low cost, and ethical and religious preferences [2].

Among the plant-based proteins, the hempseed protein isolate (HPI) [*Cannabis sativa (L.)*] has drawn increasing attention due to its nutritional qualities, high digestibility and low allergenicity [2], [3]. HPI is obtained from hempseed, a by-product of hemp, with a 50–60 % protein content after oil extraction [4]. The protein is mainly composed of edestin and albumin, which account for approximately 60–80 % and 25 % of the total protein, respectively [5]. Each edestin has six identical subunits, with one acidic subunit (AS) and one basic subunit (BS). The HPI has unfavorable solubility at neutral pH due to the high ratio of protein aggregates formed by the disulfide bonds between the AS and the BS [5], [6]. Most functional properties of a protein are only fulfilled when it is thoroughly dissolved. The inferior solubility of HPI induces poor functional properties and restricts the application of HPI in the food industry [7]. Therefore, it is imperative to seek effective methods to improve the solubility and functional properties of HPI.

Many methods have been applied to modify the natural structure of proteins to improve their functional properties, including physical, chemical, and biological technologies [8]. Among them, high-pressure homogenization (HPH) and high-intensity ultrasound (HIU) as non-thermal physical technologies have the advantages of being economical, repeatable and environmentally friendly [9], [10]. The cavitation effect is the vital mechanism of HPH and HIU during protein modification [11], [12], [13], which could generate high-speed shear force and turbulence, subsequently breaking down the intramolecular bonds of the proteins, and causing the large insoluble aggregates to dissociate into small soluble aggregates. Also, the proteins' structure could undergo alterations, improving their functional properties [14], [15]. Despite the extensive use of HIU and HPH for protein modification, the common approach in research has been to apply HIU and HPH as separate treatments, which presents certain limitations when used independently [16], [17]. For example, in cod protein treated with HPH at pressures ranging from 20 to 100 MPa, the highest increase in protein solubility achieved was only around 15 % [18]. Similarly, Tang et al., [19] found that applying HIU to Moringa oleifera seed protein at moderate amplitudes (0 %, 20 %, 40 %, and 60 %) increased water-soluble protein content from 5.56 % to 30.53 %, although foaming stability decreased from 93.94 % to 84.36 %. Further increasing the ultrasonic amplitude to 80 % caused a decline in protein solubility. Likewise, Mozafarpour et al., [20] showed that shorter HIU treatments (5 and 10 min) enhanced pea protein solubility, but extending treatment times (20 min) led to thermal degradation. Han et al., [21] compared the effects of HPH and HIU on casein, observing distinct advantages in each treatment where HPH significantly enhanced protein solubility by exposing hydrophilic areas, while HIU had a more positive effect on foamability and foaming stability.

Thus, while either HPH or HIU as a single treatment involves low processing intensity, the effects are limited and insufficient for substantial protein functionality enhancement. Increasing processing intensity could easily cause undesirable denaturation of proteins and lead to increased machine wear and tear [4].

Recognizing these limitations of the single modification technology, “dual or combined modifications” have been proposed by researchers, which refers to the application of two single modification methods in combination, presented as a two-stage modification process [22]. This approach has shown more effectiveness and lower energy requirements than the single modification process and has attracted growing attention in the food industry [23], [24].

At present, the two-stage modification combined HPH with HIU (HPH + HIU) has been applied in the whey protein isolate to improve its functional properties [23], and in the preparation of nanoemulsion to reduce the energy requirement [24]. However, limited research has applied this combined treatment to plant proteins. To the best of our knowledge, no published study has examined the effect of HIU in combination with HPH on HPI properties. This study was, therefore, performed to fill the research gap, aiming to explore the effect of HPH + HIU treatment on the changes of HPI structure and conformation, examining how this affected the solubility and other protein functional properties, including foaming and emulsifying properties, water and oil absorption capacities, and rheological behavior of HPI. Overall, the study aimed to provide fundamental knowledge to link the structure-functional relationship of HPI as influenced by HPH and HIU treatments.

## 2 Materials and methods

### 2.1 Materials

The dehulled hempseed was sourced from Hemp Connect Co., Ltd. (Levin, New Zealand). The 8-Anilino-1-naphthalenesulfonic acid (ANS), Bradford reagent and 5,5′-dithiobis (2-nitrobenzoic acid) (DTNB) were purchased from the Sigma-Aldrich (St. Louis, MO, USA). The 2 × Laemmli sample buffer, 10 × Tris/Tricine/SDS buffer, precast gel (any kD) and Coomassie brilliant blue R-250 staining solutions were obtained from Bio-Rad Laboratories, Inc. (Hercules, California, USA).

### 2.2 Hempseed protein isolate preparation

The hempseed protein isolate (HPI) was extracted following the method of Tang et al., [3] with a slight modification. The dehulled hempseed was ground and degreased twice using 10-fold (w/v) petroleum ether for 2 h. Then, the dried defatted meal was mixed with 20-fold (w/v) of pH 10.0 distilled water (pH pre-adjusted by 2 M NaOH), and stirred for 40 min at 35 °C. The mixture was centrifuged at 10,000 g for 20 min at 20 °C (Sorvall Lynx 4000, Thermo Fisher Scientific, USA). The supernatant was collected, adjusted to pH 5.0 with 2 M HCl, and stirred for 1 h. Then, the mixture was centrifuged at 10,000 g for 20 min at 4 °C, and the residue was then re-homogenized in distilled water with a ratio of 1:4. The pH was adjusted to 7.0 with 2 M NaOH. Finally, the suspension was freeze-dried (FreezeZone 12 Plus, Labconco, Kansas, MO, USA). The protein content of the HPI powder was 92.12 % (w/w dry weight), as determined using the Kjeldahl method (N × 6.25).

### 2.3 Protein modification treatments

The HPI powder was dispersed in distilled water to prepare 1 % (w/v) HPI suspension. The pH was adjusted to 7.0 using 0.1 M NaOH and stirred for 2 h. The HPI suspension was stored overnight at 4 °C to ensure sufficient hydration. The protein modification treatment was then performed as below.

The first part of the experiment was to determine the suitable pressure for protein modification. The protein solution was passed through a high-pressure homogenizer (FPG7575:S6300, Stansted Fluid Power Ltd., Essex, UK) at 60, 100, 120, 140, 180 and 200 MPa with a single cycle. The inlet and outlet homogenizing valve temperature was controlled under 40 °C by running ice water. The samples obtained after HPH treatment were immediately cooled to 4 °C by the subsidiary refrigerator. The solubility and particle size of HPI were determined (according to 2.4.1, 2.6.1), and the optimum pressure was selected for further experiments applying a two-stage modification treatment as described below.

The HPI suspension (100 mL) obtained from the optimal HPH treatment was placed into a 200 mL beaker and treated with a high-intensity ultrasound instrument (20 kHz, Sonic Ruptor 250, Omni International, Inc., Georgia, USA). The 13 mm ultrasonic probe was immersed in the HPI suspension 2 cm from the top surface. The ultrasonic treatment was performed using ultrasound power at 250 W (ultrasound intensity was 58.55 W/cm^2^ using the calorimetric method [25]). An ice bath was used to keep the sample temperature below 20 °C. After initial trials, three ultrasonic times were chosen for the investigation, i.e., 2, 5 and 10 min. The sonication was performed with 80 % pulse, meaning a pulse duration of 8 s “on” and 2 s “off”. Note that these times were chosen after preliminary experiments. According to the ultrasonic treatment time, the combination treatment was abbreviated as HPH + HIU_2min_, HPH + HIU_5min_ and HPH + HIU_10min_.

The single HPH and HIU modification treatments were also performed to provide data for comparison with the two-stage treatments. The single modification treatments were abbreviated as HPH, and HIU_2min_, HIU_5min_ and HIU_10min_ according to ultrasonic treatment time.

After the protein modification treatments, HPI samples were collected and analyzed for their physicochemical properties (according to 2.4, 2.5) and functional properties (section 2.6). The untreated HPI was used as the control.

### 2.4 Physicochemical properties

#### 2.4.1 Particle size

The particle size of HPI was measured using a Zetasizer Nano ZS instrument (Malvern, Herrenberg, Germany). The HPI solution was diluted to 0.1 mg/mL with 0.01 M phosphate buffer (pH 7.0). The absorption and refraction index of the material were set to 0.001 and 1.33, respectively [4].

#### 2.4.2 Secondary structure

The secondary structure of HPI was measured by the attenuated total reflection-Fourier transform infrared (ATR-FTIR) spectroscopy (Bruker Vertex 70, Bruker, Billerica, MA, USA). Approximately 20 µL HPI suspension (10 mg/mL) was dropped to cover the surface of the platinum-diamond. The sample scan mode was appointed as the 64 scans, the resolution was 8 cm^−1^ and the spectroscopy range was 400–4000 cm^−1^. OMNIC 4.0 and Peakfit 4.12 software were used to calculate the secondary structure content of HPI.

#### 2.4.3 Tertiary structure

##### 2.4.3.1 Intrinsic fluorescence emission

The HPI suspension was diluted to 0.2 mg/mL with 10 mM sodium phosphate buffer (pH 7.0). The fluorescence intensity (FI) was measured using the plate reader (EnSpire Multimode, Perkin Elmer, Waltham, MA, USA) with a fluorescence spectrum mode at the excitation wavelength of 280 nm and emission wavelength of 300–400 nm.

##### 2.4.3.2 Surface hydrophobicity

The surface hydrophobicity (H_0_) of HPI was determined by using 1-anilino naphthalene-8-sulfonic acid (ANS) as the fluorescent probe (Karabulut & Yemiş, 2022). Briefly, 3 mL of HPI suspension (1 mg/mL) was incubated with 20 μL 8 mM ANS for 25 min in the darkness. Then, the mixture was read at the excitation wavelength of 300 nm, and emission wavelength of 410–570 nm with a 5.0 nm slit (EnSpire Multimode, Perkin Elmer, Waltham, MA, USA).

##### 2.4.3.3 Total and free sulfhydryl contents

The total sulfhydryl and free sulfhydryl contents were determined according to Mao et al. [26]. The 0.5 mL HPI solution (1 mg/mL) was mixed with 5 mL sulfhydryl buffer (86 mM tris, 90 mM Gly, 4 mM EDTA-Na_2_, 8 M urea, pH 8.0) and 100 µL DTNB solution. The mixture was incubated at 25 °C for 30 min without lightness. The free sulfhydryl content was determined as 1 mL of HPI solution (1 mg/mL) mixed with 100 µL DTNB solution. The absorbance of the mixture was read at 412 nm (EnSpire Multimode, Perkin Elmer, Waltham, MA, USA).

#### 2.4.4 Sodium dodecyl sulfate–polyacrylamide gel electrophoresis (SDS-PAGE)

The HPI suspension (4 mg/mL) was mixed with an equal volume of 2 × SDS Laemmli sample buffer (with or without 5 % β-mercaptoethanol (βME), and the mixture was boiled at 95 °C for 5 min. Then, 11 µL samples were loaded into the precast gel and ran at 80 V until the bromophenol blue reached the bottom of the gel. After electrophoresis, the gels were stained with Coomassie brilliant blue solution for 60 min and subsequently destained with 10 % methanol and 10 % acetic acid overnight.

### 2.5 Microstructure

#### 2.5.1 Scanning electron microscopy

The lyophilized HPI powder was stuck in the copper platform and observed using scanning electron microscopy (SEM). The accelerated voltage was 10 kV, and the magnification was 10,000× (SU-70, Hitachi, Tokyo, Japan).

#### 2.5.2 Atomic force microscopy

The HPI suspension (0.1 mg/mL) was dropped on the surface of the fresh mica (5 × 5 cm^2^) and dried by airflow. The samples were then observed using an atomic force microscopy (AFM) instrument (Cypher-ES, Asylum Research, USA) equipped with a silicon AFM probe (Multi75Al-G, Budget Sensors Ltd., Sofia, Bulgaria). The scan size was set as 5 μm, and the scan rate was 1.50 Hz. The AFM images were visualized and analyzed using the software Gwyddion 2.63.

### 2.6 Functional properties

#### 2.6.1 Solubility

The HPI suspension (2 mg/mL, pH 7.0) was centrifuged at 10,000 g for 10 min at 4 °C (Sorvall Lynx 4000, Thermo Fisher Scientific, USA). The protein content was determined using the Bradford reagent with the bovine serum albumin as standard. The solubility was calculated as the percentage of the protein content in the supernatant to the total protein content before centrifugation [7].

#### 2.6.2 Water and oil absorption capacity

The HPI powder (20 mg) was dispersed in 600 µL distilled water and soybean oil. After evenly vortexing for 1 min, the mixture was kept standing for 30 min at room temperature and then centrifuged at 6,000 g for 10 min at 4 °C. The tube was inverted to remove the supernatant. The water and oil absorption capacity (WAC and OAC) were determined from the weight difference between the final tube contents and the initial HPI powder [4].

#### 2.6.3 Foaming capacity and foam stability

The HPI powder was dispersed in the 10 mM phosphate buffer (pH 7.0) and the concentration was adjusted to 10 mg/mL. Then, 5 mL HPI suspensions were homogenized at 16,000 rpm for 2 min to form the foam (Ultra-Turrax T-25, IKA Instruments, Germany). The foaming capacity (FC) was calculated as the percentage of the volume of the initial foam related to the 5 mL HPI suspensions. The foam stability (FS) was calculated as the percentage of the foam after standing for 30 min compared to the 5 mL HPI suspensions [16].

#### 2.6.4 Emulsion activity and stability

The emulsion activity index (EAI) and emulsion stability index (ESI) of HPI were determined by a slight modification of Yang et al. [16] 's methods. Four mL HPI suspension (10 mg/mL) was homogenized with 1 mL soybean oil for 1 min at 22,000 rpm (Ultra-Turrax T-25, IKA Instruments, Germany). Subsequently, 50 µL emulsion was taken from the bottom of the homogenate and diluted 100-fold with 0.1 % SDS solution. EAI was determined by reading the absorbance at 500 nm of the diluted emulsion. ESI was determined using the same method after allowing the emulsion to stand for 10 min. EAI and ESI were calculated using Eq (1), (2).

(1) $$EAI\;\left(m2/g\right)\;=\;\frac{2*2.303*A_{0}*DF}{C*\Phi *\theta *1000}$$

(2) $$ESI\;\left(min\right)\;=\;\frac{A_{0}*\Delta_{t}}{A_{0}-A_{10}}$$

Where DF is the dilution factor, C is the initial HPI concentration (g/mL), Φ is the optical path, and θ is a portion of oil used (mL/mL), whereas A_0_ and A_10_ are the absorbances of the diluted emulsions after 0 min and 10 min, respectively.

#### 2.6.5 Rheological properties of HPI emulsion

The HPI emulsion prepared in Section 2.6.4 was applied on the platform without bubbles and equilibrated for 30 s at 25 °C (PP50, MCR 302, Anton Paar, Austria), then sheared along with the shear rate increased from 1 to 100 s^−1^ [14].

### 2.7 Statistical analysis

Each treatment was repeated four times. The data were analyzed using one-way ANOVA with Duncan's multiple-range tests. The results were expressed as the means ± standard errors. The difference was deemed significant when *P* < 0.05. The Pearson's correlation and Principal Component Analysis (PCA) were performed using the Origin2021Pro (OriginLab Co., Northampton, MA).

## 3 Results and discussion

### 3.1 Optimization of the pressure for HPH

Numerous studies have reported that the protein's solubility is closely related to their particle size and polydispersity index (PDI). Smaller particle size and uniform dispersion system could increase the surface area of proteins in contact with the water, which enhances the protein-water interactions and, in turn, increases the solubility [15], [27]. Therefore, the solubility, particle size and PDI of HPI were chosen as the optimal indicators in the current study.

**Fig. 1:**
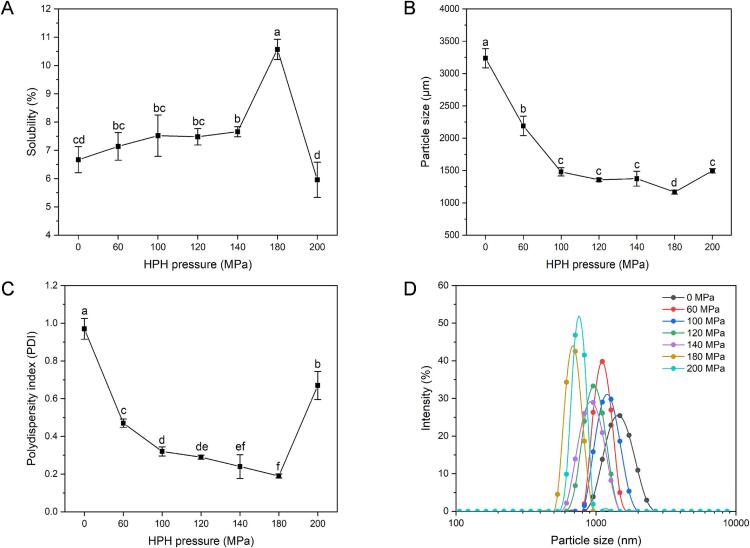
Properties of hempseed protein isolate (HPI) under different pressure treatments. A: Solubility; B: Particle size; C: Polydispersity index (PDI); D: Particle size distribution. Different letters (a-d) indicate statistically significant differences (*P* < 0.05).

Fig. 1A shows the solubility of HPI after different pressure treatments. The solubility of untreated HPI obtained in this study was 7.52 % at neutral pH, consistent with previous research for HPI [3], [28]. Treatments at low pressure (60, 100 and 120 MPa) did not induce a significant change in solubility (*P* > 0.05). When the pressure increased to 140 MPa, the solubility significantly increased to 7.66 % (*P* < 0.05). Subsequently, it reached the maximum value (10.57 %) when the pressure was 180 MPa (*P* < 0.05). The solubility then decreased to 5.96 % when the pressure rose to 200 MPa, which might be related to the protein re-aggregation caused by the “over-processing” effect [14]. Nevertheless, the increase in solubility due to increasing pressure from 0 MPa to 180 MPa was not very high, only 40.56 %, indicating the limitation of the single HPH treatment.

From Fig. 1B, the particle size of the HPI decreased substantially with the pressure treatment from 0 to 180 MPa, with the minimum particle size observed at 180 MPa (*P* < 0.05). When the pressure increased to 200 MPa, the particle size was reversibly increased compared to that of the 180 MPa treatment (*P* < 0.05). These results indicate that the larger insoluble aggregates were broken down into smaller aggregates under moderate pressure. On the other hand, smaller aggregates could reform into larger aggregates at a higher pressure, as reflected at 200 MPa. The PDI (Fig. 1C) exhibited similar trends with the particle size. When the pressure increased from 60 to 180 MPa, the PDI significantly decreased until it reached the lowest value at 180 MPa (Fig. 1D) (*P* < 0.05), indicating narrow particle size distribution and particle homogeneity [29]. However, when the pressure increased to 200 MPa, the PDI became larger than the 180 MPa treatment (*P* < 0.05), consistent with the particle size result. This observation suggests that treatment above 180 MPa could lead to more unstable dispersion of HPI. Overall, the results indicated that a homogenization pressure of 180 MPa was optimum for enhancing the solubility and dispersion behavior of HPI, and it was selected for further experiments.

### 3.2 Solubility and particle size

**Fig. 2:**
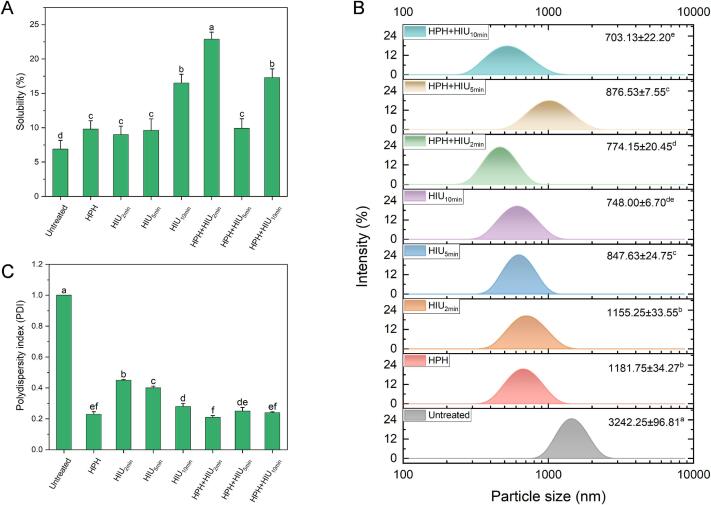
Dispersion behavior of hempseed protein isolate (HPI) after single (HPH or HIU) and two-stage treatments (HPH + HIU). A: Solubility; B: Particle size distribution; C: Polydispersity index (PDI). Columns with different letters (a-d) indicate statistically significant differences (*P* < 0.05). HPH: high-pressure homogenization treatment; HIU: high-intensity ultrasound treatment; HPH + HIU: high-pressure homogenization coupled with high-intensity ultrasound treatment.

Fig. 2 displays the change of solubility, particle size distribution, and PDI of HPI under different single HPH or HIU treatments and the combined two-stage treatments (HPH + HIU treatments). Compared to the untreated HPI, all the single modification treatments of HPH and HIU significantly improved the solubility (*P* < 0.05) (Fig. 2A).

For the two-stage treatments, HPH combined with shorter ultrasonic time (i.e., HPH + HIU_2min_) could effectively enhance the HPI's solubility to the highest value (*P* < 0.05), showing a 232.7 % improvement compared to the untreated HPI. In comparison, HPH combined with a longer ultrasonic time (i.e., HPH + HIU_5min_ and HPH + HIU_10min_ treatments) caused lower solubility compared to the HPH + HIU_2min_ treatment (*P* < 0.05).

The HPI's particle size (Fig. 2B) significantly decreased after all the treatments compared to the untreated HPI. Specifically, extending the ultrasonic time to 5 and 10 min for the HIU treatment reduced the particle size (*P* < 0.05), with the peak of the particle size distribution shifted to a smaller size. This finding could be attributed to the strengthened cavitation effect due to the longer sonication, which could disintegrate the macromolecular aggregates into smaller aggregates.

For the two-stage treatments (Fig. 2B), all three treatment combinations gave significant changes in particle size among the samples (*P* < 0.05). Specially, compared to the HPH and HIU_2min_, the HPH + HIU_2min_ treatments showed smaller particle size (*P* < 0.05), which indicated the synergistic effect of HPH and HIU on disintegrating the large protein aggregates to form the smaller protein particles. Furthermore, it increased the specific surface area of HPI interacting with water molecules, resulting in higher HPI solubility in the HPH + HIU_2min_ treated samples. When the ultrasonic time increased from 2 min to 5 min, the particle size and PDI increased (Fig. 2C) (*P* < 0.05). However, further extension of ultrasonic time to 10 min decreased the particle size (*P* < 0.05). This result indicated that the ultrasonic treatment of 5 min could cause the re-forming of larger protein aggregates and induce instability in HPI dispersion. In contrast, extending ultrasonic time to 10 min (i.e., HPH + HIU_10min_ treatment) could alleviate this phenomenon as the smaller particle size was observed.

### 3.3 Protein conformation

#### 3.3.1 Secondary structure

**Fig. 3:**
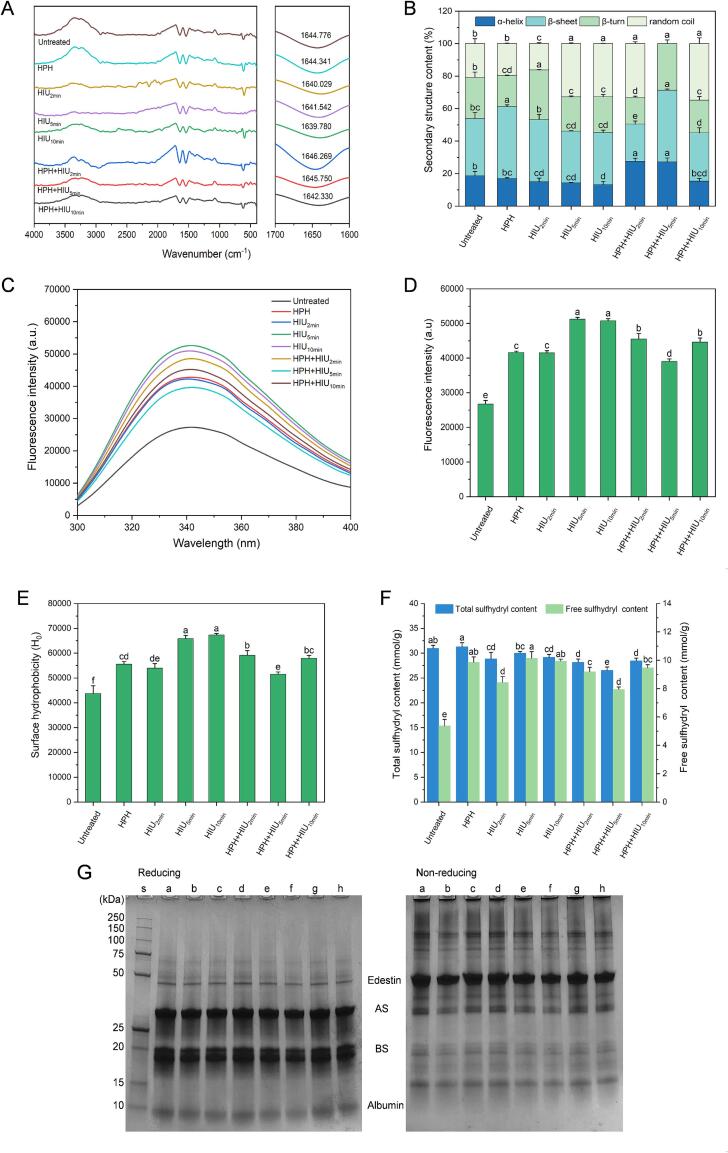
Physicochemical properties of hempseed protein isolate (HPI) after single (HPH or HIU) and two-stage (HPH + HIU) treatments. A: FTIR spectra; B: Secondary structure content; C: Intrinsic fluorescent spectra; D: Fluorescence intensity; E: Surface hydrophobicity; F: Total and free sulfhydryl content; G: SDS-PAGE. Columns with different letters (a-d) indicate statistically significant differences (*P* < 0.05). HPH: high-pressure homogenization treatment; HIU: high-intensity ultrasound treatment; HPH + HIU: high-pressure homogenization coupled with high-intensity ultrasound treatment. s: standard marker; a: Untreated HPI; b: HPH; c: HIU_2min_; d: HIU_5min_; e: HIU_10min_; f: HPH + HIU_2min_; g: HPH + HIU_5min_; h: HPH + HIU_10min_.

FTIR is a vital indicator for evaluating proteins' secondary structure and chemical bonds. Fig. 3A shows the FTIR spectrum of HPI under different treatments. The secondary structure content of HPI was analyzed from the amide I band (1700–1600 cm^−1^) [30]. For the untreated HPI, the transmission peak was located at 1644.776 cm^−1^, with the main secondary structure consisting of β-sheet (35.30 %), followed by the β-turn (25.27 %), random coil (20.78 %) and α-helix (18.65 %) (Fig. 3B). These results are consistent with the research of [31]. The high β-sheet content might be one reason for the compact structure of HPI, which was responsible for the poor solubility at neutral pH [2]. In this study, the HPH treatment induced more β-sheet structure formed compared to the untreated HPI (*P* < 0.05), while the HIU treatment did not significantly change the β-sheet content compared to the untreated HPI (*P* > 0.05). However, it is noted that when applied in combination with HPH, a shorter ultrasonic time of 2 min (i.e., HPH + HIU_2min_) was capable of reducing the β-sheet content to the lowest level among all the treatment groups (*P* < 0.05). This clearly demonstrated that the combined treatment of HPH + HIU_2min_ could exert a superior effect in disrupting the intermolecular hydrogen bonds and reducing the β-sheet content than the single treatment of HPH and HIU_2min_. In addition, the α-helix content in the HPH + HIU_2min_ treatment group was increased to a higher level than that of the untreated HPI and the HPH and HIU treatment groups (*P* < 0.05), as the transmittance peak shifted to the larger wavenumber (1646.269 cm^−1^). Furthermore, the random coil content was higher after the HPH + HIU_2min_ treatment than after the HPH and HIU_2min_ treatments (*P* < 0.05). These changes in the secondary structure indicated that the compact protein structure was unfolded and stretched [32], which might consequently lead to the internal hydrophobic regions being exposed to the surface of the proteins.

Compared with the treatment at longer ultrasonic treatment, the β-sheet content observed after the HPH + HIU_5min_ treatment was inversely increased compared to the HPH + HIU_2min_ treatment (*P* < 0.05). Moreover, the random coil structure was not detected. These results indicated that the HPI was refolded and reversibly converted to a more ordered structure upon the HPH + HIU_5min_ treatment, and the protein aggregates were reformed [17]. While increasing the ultrasonic time to 10 min (i.e., HPH + HIU_10min_ treatment), the HPI was transformed into a more flexible secondary structure as the β-sheet content decreased, and the random coil content increased compared to the HPH + HIU_5min_ treatment (*P* < 0.05).

#### 3.3.2 Tertiary structure

Protein possesses chromogenic amino acids, especially the tryptophan residues, which have inherent fluorescence and are sensitive to the polarity of the microenvironment. This aspect could be applied to evaluate the tertiary structure of proteins. The intrinsic fluorescence spectra of HPI under different treatments are presented in Fig. 3C. The maximum emission wavelength (λ_max_) of all the treatment groups was maintained at 340 nm without any significant shift. Fig. 3D shows the fluorescence intensity (FI) of HPI. After treatments, the FI was remarkably increased compared to the untreated HPI (*P* < 0.05). This finding indicated the changes in the HPI conformation where more previously buried tryptophan residues in the internal non-polar region were exposed to HPI's surface [33]. Notably, the FI of the HPH + HIU_2min_ treatment group was higher than that of the HPH and HIU_2min_ treatment groups (*P* < 0.05). The results showed that combining HPH and a shorter ultrasonic treatment could have a synergetic effect to expand the HPI structure and bring more tryptophan residues to the external environment. However, the HPH + HIU_5min_ and HPH + HIU_10min_ treatments significantly decreased the FI compared to the HIU_5min_ and HIU_10min_ treatments (*P* < 0.05). It suggests that increasing the ultrasonic time of HIU for the combination treatment could lead to protein aggregation and reburial of chromogenic amino acid residues [17].

The surface hydrophobicity (H_0_) was also used to depict the protein tertiary structure based on the interaction of ANS with the surface hydrophobic region of protein through non-covalent bonds [33]. The results of H_0_ (Fig. 3E) showed similar trends to those of FI. Generally, the findings revealed that HPH and HIU treatments could increase the H_0_ compared to the untreated HPI (*P* < 0.05), indicating that the internal hydrophobic groups were exposed to the HPI surface due to the protein unfolding. Notably, it was evident that after the two-stage HPH + HIU_2min_ treatment, the H_0_ of HPI gave a more remarkable improvement than the single HPH or HIU_2min_ treatments (*P* < 0.05). The results further indicated the synergistic effect of HPH and HIU that caused more intense stretching and unfolding of the protein structure. Furthermore, a smaller particle size obtained after the HPH + HIU_2min_ treatment indicated that the insoluble aggregates were most severely disrupted, thus exposing more hydrophobic regions. In addition, for the single HIU treatment, it was noted that when the ultrasonic time was extended to 5 and 10 min, the H_0_ of the HIU_5min_ and HIU_10min_ treatment group was significantly increased compared to that obtained from the HIU_2min_ treatment (*P* < 0.05). It could be due to the more intense cavitation resulted from the longer sonication, which could further unfold the protein molecule. However, increasing the ultrasonic time to 5 and 10 min for the combination treatment caused the H_0_ to drop to a lower level compared to the single process of HIU_5min_ and HIU_10min_ treatments (*P* < 0.05). This indicated that extending the ultrasonic time induced an “over-processing” effect for the two-stage HPH + HIU combination treatment. The excessive energy could lead the previously exposed hydrophobic groups to interact with each other through the hydrophobic interaction, thus reburying the hydrophobic groups.

#### 3.3.3 Total and free sulfhydryl content

The total and free sulfhydryl content could reflect the alteration in the tertiary protein structure and may be closely related to protein functionality. In addition, the edestin of HPI has abundant cysteine residues, which have a high content of highly reactive sulfhydryl groups, making the protein prone to aggregate through the disulfide bonds [5]. Therefore, it is valuable to estimate the total and free sulfhydryl content of HPI.

Fig. 3F shows the results of the total and free sulfhydryl content of HPI under different treatments. Compared to the untreated HPI, a significant increase in the free sulfhydryl content was observed after all the treatments, regardless of the methods used (*P* < 0.05). This phenomenon could be related to the disaggregation of larger protein aggregates, which exposed the previously embedded sulfhydryl groups to the protein surface [34]. In addition, the HPH and HIU treatments could generate mechanical effects such as shear force and micro-steaming that disrupted the disulfide bonds of protein aggregates, promoting the formation of new free sulfhydryl groups [20].

Based on the above results, one may infer that the two-stage combination treatment will give a higher free sulfhydryl content. However, contrary to the inference, the HPH + HIU_2min_ and HPH + HIU_5min_ treatments gave lower free sulfhydryl contents than those of the single HPH, HIU_5min_ and HIU_10min_ treatments (*P* < 0.05). As reported in the literature, HPH and HIU treatments could hydrolyze water molecules by the cavitation effects and generate highly reactive free radicals, consequently oxidizing the susceptible sulfhydryl groups to form disulfide bonds [27], [35]. In the two-stage treatments, i.e., the HPH + HIU_2min_ and HPH + HIU_5min_ treatments, the cavitation effects might be strengthened due to combined treatment. As a result, more highly reactive free radicals were generated, thus consuming more sulfhydryl groups and resulting in lower free sulfhydryl content. However, when the ultrasonic treatment was prolonged to 10 min (i.e., HPH + HIU_10min_ treatment), the free and total sulfhydryl content increased again compared to the HPH + HIU_5min_ treatment (*P* < 0.05). This showed that the formation and breakage of disulfide bonds could change dynamically depending on the treatment conditions. When HPI was subjected to single HPH, HIU and moderate two-stage modification treatments (i.e., HPH + HIU_2min_), the intermolecular disulfide bonds were broken as the protein structure unfolded. As the cavitation effect was reinforced with longer ultrasonic time as in the HPH + HIU_5min_ treatment, the exposed sulfhydryl groups tended to interact, and the oxidation effect also induced the protein to aggregate, resulting in new disulfide bonds. After applying a more robust cavitation effect (HPH + HIU_10min_), the destructive effect on protein aggregates was more substantial than the promoting effect on protein aggregates, causing the protein aggregates to partially unfold again. Notably, the transformation of the secondary structure and the increase in FI and H_0_ in the HPH + HIU_10min_ treatment group confirmed the change in the protein structure.

#### 3.3.4 SDS-PAGE

Fig. 3G shows the protein subunits of HPI obtained with SDS-PAGE under the reducing and non-reducing conditions. The reducing band pattern obtained in this study coincided with a previous study by Shen, et al., [36]. The 50 kDa band could be identified as edestin, and the 15–10 kDa band was albumin [6]. As stated above, each edestin subunit contains an AS (33 kDa) and a BS (20 kDa), and they are cross-linked by the disulfide bonds [36]. Under the reducing condition, βME could disrupt the disulfide bonds and, thus, disintegrate edestin. This is reflected in a weakened 50 kDa band and the deepened bands of AS and BS on the SDS-PAGE. There were no significant changes in the overall band patterns among all the treatments, which indicated that none of the modification treatments could alter the primary structure of HPI.

### 3.4 Microstructure

**Fig. 4:**
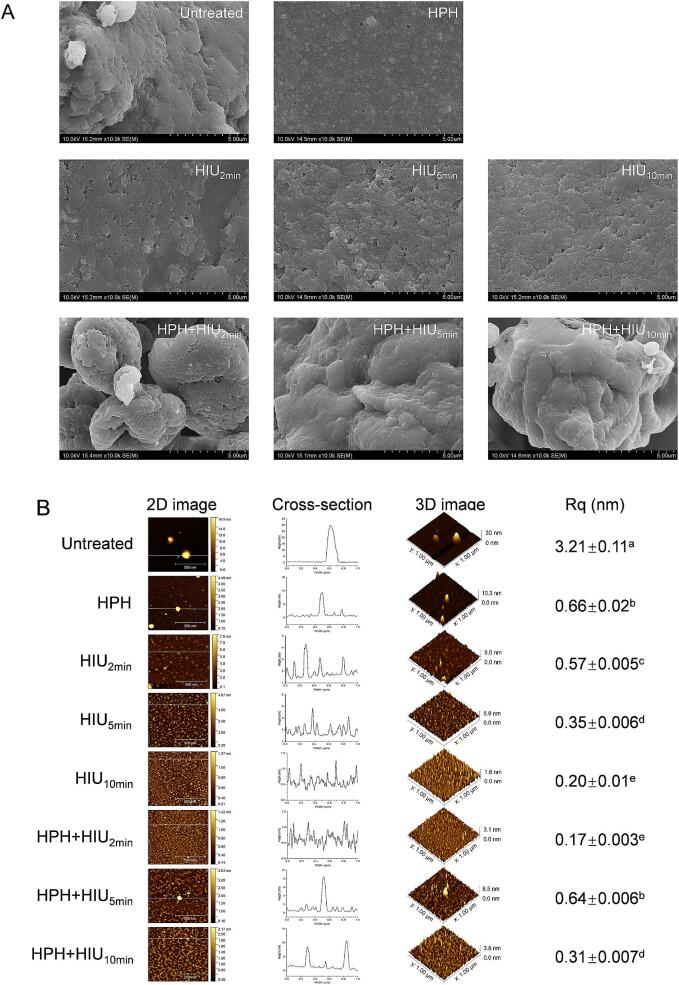
Microstructure of hempseed protein isolate (HPI) after single (HPH or HIU) and two-stage (HPH + HIU) treatments. A: Scanning electron microscopy (SEM); B: Atomic force microscopy (AFM). Different letters (a-d) indicate significant statistical differences (*P* < 0.05). HPH: high-pressure homogenization; HIU: high-intensity ultrasound; HPH + HIU: high-pressure homogenization coupled with high-intensity ultrasound.

Fig. 4A shows the surface microstructure of HPI powders observed using the SEM. The untreated HPI powder displayed a smooth and compact structure and typical plate-shaped pattern due to lyophilization, which is in agreement with the findings of Fang et al. [31]. After the HPH and HIU treatments, the surface of HPI powder became rough, accompanied by the formation of irregular aggregates. In addition, an enormous number of pores were observed on the surface, which could improve the accessibility of water molecules to the HPI.

Notably, the surface microstructure change became more apparent after the HPH + HIU_2min_ treatment. The plate-shaped structure was transformed entirely into the agglomerate aggregate with a loose structure. This change increased the surface area of the HPI and provided channels for water molecules to enter the inner protein structure, resulting in the highest HPI solubility, as observed earlier (Fig. 2A). However, when subjected to the HPH + HIU_5min_ treatment, the HPI microstructure reversed to the plate shape with a compact structure, with no visible pore being found. It confirmed the formation of protein aggregation, as described in the protein particle size distribution and protein conformation. After more prolonged ultrasonic treatment, i.e., the HPH + HIU_10min_ treatment, the compact microstructure was partially loosened, as shown by the agglomerated aggregate without pores. These results further demonstrated that more substantial cavitation could partially destroy the protein aggregates formed in the HPH + HIU_5min_ treatment.

Next, AFM was applied to detect the surface morphology of HPI at the nanoscale and reflect the three-dimensional information. As shown in Fig. 4B, the untreated HPI exhibited spherical aggregation with the highest cross-section height (30 nm) and relatively most significant roughness (3.21) among all samples (*P* < 0.05). This observation further confirmed the presence of large protein aggregates that explained the inferior solubility of the untreated HPI. After the HPH and HIU treatments, the surface roughness (Rq) was reduced compared to the untreated HPI (*P* < 0.05), and the cross-section height tended to be more evenly distributed. However, larger aggregates could still be detected in the HPH and HIU_2min_ groups, indicating that the single treatment only limitedly broke down the insoluble aggregate.

After the HPH + HIU_2min_ treatment, the height of the aggregates and Rq decreased compared to the HPH, HIU_2min_ and HIU_5min_ treatments (*P* < 0.05). The results show a uniform decrease in protein aggregate size, as evidenced by the 2D imaging and consistent cross-sectional height distribution. This indicates that the two-stage combination of HPH and HIU_2min_ treatment could synergistically dissociate the larger protein aggregates into smaller soluble aggregates, leading to the more uniform dispersion of HPI. Consequently, this treatment facilitates the HPI to achieve the highest solubility in the HPH + HIU_2min_ group.

When the ultrasonic treatment time increased to 5 min (i.e., HPH + HIU_5min_ treatment), Rq increased significantly compared to the HPH + HIU_2min_ treatment (*P* < 0.05), which corresponded to the results of particle size, further confirming the formation of HPI aggregates in the samples. Protein structure returned to the compact state, and the sites for protein interaction with the water molecules could be reburied in the inner structure, reducing the HPI solubility.

However, with further increasing ultrasound treatment to 10 min (HPH + HIU_10min_ treatment), the Rq reduced, which might be resulted from the highest cavitation intensity that could lead to partial disruption of HPI aggregates in the samples. In addition, according to the higher value of FI and H_0_ in the HPH + HIU_10min_ treatment groups, the active groups were partially exposed to the protein surface again. It facilitated recovery of the HPI solubility, thus leading to a higher solubility than that in the HPH + HIU_5min_ treatment groups.

### 3.5 Functional properties

#### 3.5.1 Water and oil absorption capacities (WAC/OAC)

**Fig. 5:**
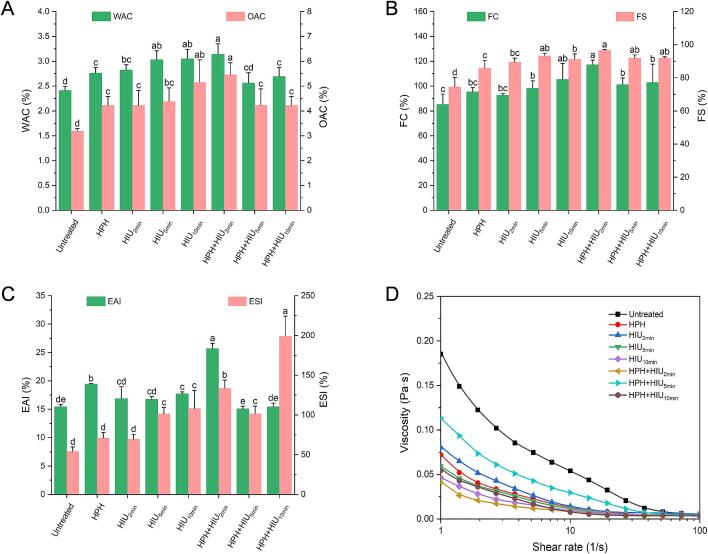
Functional properties of hempseed protein isolate (HPI) after single (HPH or HIU) and two-stage (HPH + HIU) treatments. A: Water and oil absorption capacity (WAC/OAC); B: Foaming capacity (FC) and foam stability (FS); C: Emulsion activity index (EAI) and emulsion stability index (ESI); D: Viscosity-shear rate of HPI emulsions. Columns with different letters (a-d) indicate significant statistical differences (*P* < 0.05). HPH: high-pressure homogenization treatment; HIU: high-intensity ultrasound treatment; HPH + HIU: high-pressure homogenization coupled with high-intensity ultrasound treatment.

WAC and OAC imply the amount of water or oil that can be absorbed per gram of protein, respectively, which are closely related to the shelf-life, texture, emulsifying properties, flavor and other quality attributes of food products [4], [30]. As shown in Fig. 5A, the single HPH treatment led to a significant enhancement in the WAC from 2.41 to 2.76 % (*P* < 0.05), while the HIU treatment increased the WAC to 2.82, 3.03 and 3.05 %, corresponding to the ultrasonic time of 2, 5, and 10 min (*P* < 0.05). The results illustrated that the HPH and HIU treatments could improve the protein-water interactions and facilitate better entrapment of water in the protein matrix.

The WAC of HPI increased remarkedly after the two-stage HPH + HIU_2min_ treatment compared to those obtained after single HPH or HIU_2min_ treatments, reaching the value of 3.14 % (*P* < 0.05). However, the WAC value decreased to 2.56 and 2.69 after the HPH + HIU_5min_ and HPH + HIU_10min_ treatments, respectively. The OAC of HPI showed similar trends to that of the WAC after various treatments. The maximum OAC value was achieved after the HIU_10min_ and HPH + HIU_2min_ treatments (*P* < 0.05). These results further revealed that the HPH + HIU_2min_ treatment was efficient in improving the OAC and WAC of HPI, which could be ascribed to the better hydrophilic/hydrophobic balance of HPI molecules in the HPH + HIU_2min_ treatment group. Along with the loose structure caused by the HPH + HIU_2min_ treatment, which allowed more hydrophilic sites to bind the water molecules, it also exposed more hydrophobic groups on the outer structure that could interact with more oil molecules. Furthermore, from the SEM and particle size results, the structure of HPI powder of HPH + HIU_2min_ treatment showed superiority in the surface area, which might explain the increment in the WAC and OAC [4].

#### 3.5.2 Foaming properties

Fig. 5B shows the foaming capacity (FC) and foam stability (FS) of HPI after different treatments. The results revealed that the HPH and HIU_2min_ treatments did not cause a significant change in the FC (*P* > 0.05). By increasing the ultrasonic time to 5 and 10 min for the HIU treatment, the FC was considerably raised from the native value of 85.17 % to 98.22 % and 105.42 % due to the more substantial cavitation effect (*P* < 0.05). Furthermore, applying the HPH + HIU_2min_ treatment could enhance the FC to a level similar to that of the HIU_10min_ treatment (*P* > 0.05). However, increasing the ultrasonic time to 5 and 10 min in the combined treatment (HPH + HIU_5min_ and HPH + HIU_10min_) led to a decrease in FC compared to the HPH + HIU_2min_ treatment (*P* < 0.05). Han et al. [21] suggested that this decline could be attributed to protein aggregation, which could inhibit molecular mobility and limit foam formation.

On the other hand, the FS significantly increased by all treatments (*P* < 0.05). The HPH + HIU_2min_ treatment improved the FC more than the single HPH and HIU_2min_ treatments (*P* < 0.05). This could be attributed to the highest protein solubility after the HPH + HIU_2min_ treatment, which allowed the most significant amount of protein available to encapsulate air for foam formation. Also, the loose protein structure, as observed in the protein conformation and microstructure (3.3, 3.4), allowed better diffusion and protein adsorption at the air–liquid interface to stabilize foam, facilitating the formation of a cohesive and strong layer to hinder foam deformation [37].

#### 3.5.3 Emulsifying properties

Fig. 5C shows the EAI and ESI of HPI after different treatments. The finding revealed that a single HPH treatment at 180 MPa could significantly increase the EAI compared to the untreated HPI (*P* > 0.05). However, the single modification HIU treatment showed no significant effect on the EAI unless a longer ultrasonic time of 10 min was applied. It was apparent that the HPH + HIU_2min_ treatment caused the EAI to increase to the maximum value compared to other treatments (*P* < 0.05). Specifically, the EAI after the HPH + HIU_2min_ treatment improved by 32.25 % and 33.98 % compared to the single HPH and HIU_2min_ treatments. Nevertheless, with longer ultrasonic treatments (i.e., HPH + HIU_5min_ and HPH + HIU_10min_ treatment), the EAI declined significantly compared to the HPH + HIU_2min_ treatment (*P* < 0.05).

For the ESI, the single HPH treatment and short ultrasound treatment at 2 min (HIU_2min_) did not cause any significant change compared to the untreated HPI (*P* > 0.05). In contrast, a two-stage modification process of HPH + HIU_2min_ treatment increased the ESI by 146.84 % from the untreated HPI value. In fact, the ESI of the HPH + HIU_2min_ treatment was significantly higher than those obtained from all treatments except the HPH + HIU_10min_ treatment (*P* < 0.05). A closer look at the results revealed that after the HPH + HIU_5min_ and HPH + HIU_10min_ treatments, the ESI was inversely decreased and increased again compared to the HPH + HIU_2min_ treatment (*P* < 0.05). This result might be caused by protein aggregation and partial re-folding due to more extended ultrasonic treatment during the HPH + HIU_5min_ and HPH + HIU_10min_ treatment stages.

Overall, the results of EAI and ESI indicated that the HPH + HIU_2min_ treatment was more effective in improving the emulsifying properties of HPI. The highest HPI solubility obtained after the HPH + HIU_2min_ treatment might be responsible for its superiority, as sufficient protein dissolution in water could facilitate its rapid dispersion in the oil–water interface and form a dense protein film to cover the oil droplet surface. On the other hand, more exposed hydrophobic groups enhanced the possibility of HPI adsorption at the oil–water interface to lower the interfacial tension, thus improving the EAI and ESI of HPI.

#### 3.5.4 Rheological properties of HPI emulsion

Fig. 5D shows the viscosity change along with the shear rate, reflecting the protein interaction and resistance to the shear. The results exhibited that all HPI samples showed shear-thinning behavior, as the viscosity decreased with the increase in shear rate [38] regardless of treatments. However, after treatments, the viscosity flow curves were significantly lower than that of the untreated HPI emulsion. This phenomenon might be ascribed to the smaller particle size in the treated samples, which could reduce the protein entanglements and internal friction. As a result, it could promote the HPI molecules to orient rapidly along with the shear force, thus decreasing the viscosity [14], [39].

It was noteworthy that among all treatments, the HPH + HIU_2min_ treatment gave the lowest viscosity, indicating that the HPH and shorter ultrasonic time could synergistically increase the fluidity of the HPI emulsion. When ultrasonic time increased to 5 min in the HPH + HIU_5min_ treatment, the viscosity increased to a higher level than other treatments, most probably due to protein re-aggregation, promoting higher resistance to external shear force. The effect was reduced with more prolonged ultrasonic treatment (HPH + HIU_10min_) as protein aggregates partially dissociated and the protein connection was impaired.

### 3.6 Correlation analysis and principal component analysis

**Fig. 6:**
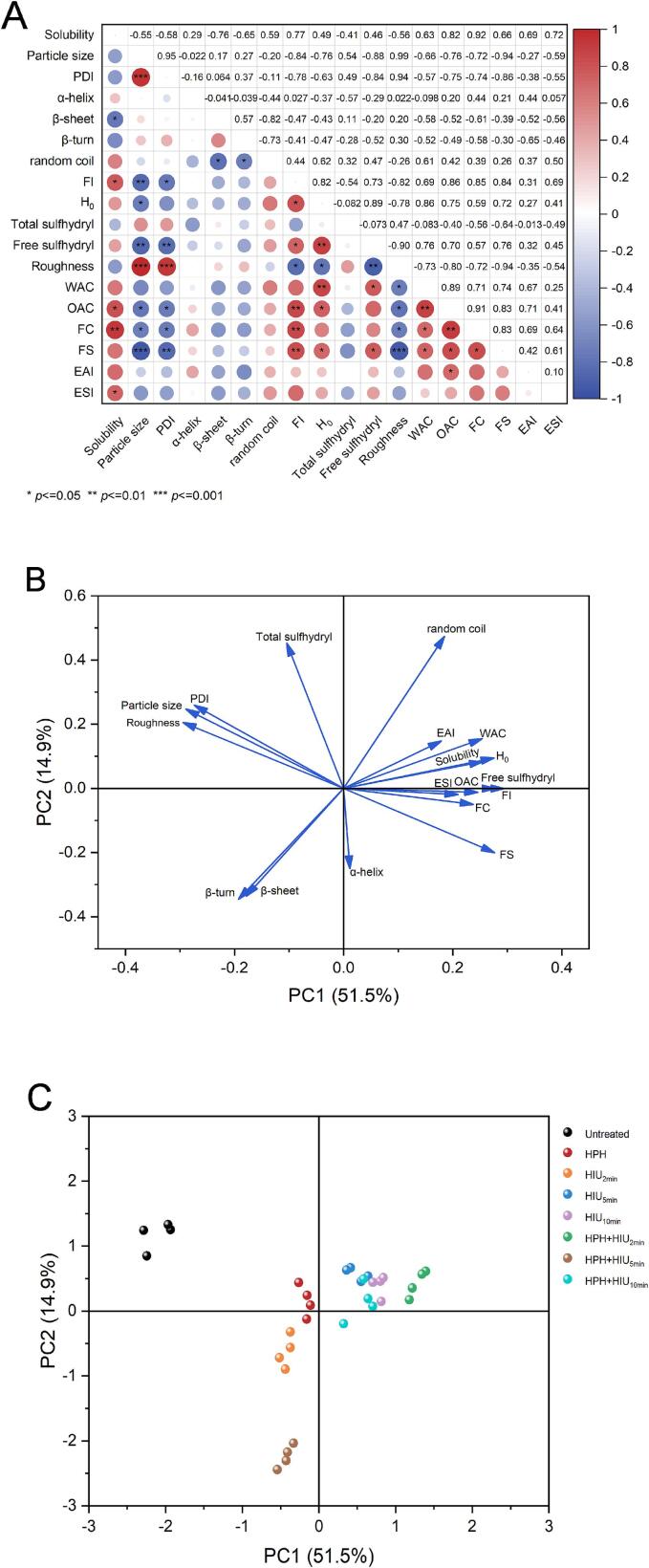
Pearson’s correlation and Principal Component Analysis (PCA) of physicochemical and functional properties of HPI under single and combined treatments. A: heatmap of Pearson’s correlation analysis; B: PCA loading plots; C: PCA factor score plots. HPH: high-pressure homogenization treatment; HIU: high-intensity ultrasound treatment; HPH + HIU: high-pressure homogenization coupled with high-intensity ultrasound treatment.

Correlation analysis and the principal component analysis (PCA) were performed to investigate the relationship between the physicochemical and functional properties of HPI and provide a better understanding of the effects of single and combined treatments. Fig. 6A shows the correlation heat map where the color changed from blue to red and the circle changed from small to large, indicating a more positive and stronger correlation. It was obvious that the particle size, PDI, surface roughness and the β-sheet content were negatively correlated with the functional properties of HPI. In contrast, the FI, H_0_, free sulfhydryl content and the functional properties of HPI had a positive relationship. These findings further confirmed that the compact structure of HPI was not conducive to fulfilling its functionality, while the unfolded or partially protein structure obtained after the single (HPH or HIU) and two-stage modification treatments (HPH + HIU) could improve its functionality.

The PCA loading plots (Fig. 6B) further support the findings in Fig. 6A. The PC1 (51.5 %) showed positive interrelations with the random coil content, EAI, ESI, WAC, OAC, FC, FS, solubility, FI, H_0_ and free sulfhydryl content of HPI. The PC2 (14.9 %) had negative correlations with the β-sheet and β-turn content of HPI. In Fig. 6C, the single and two-stage modification treatments were significantly distinguished from the untreated treatment. The results also indicated that both the single and two-stage modification treatments had impacted the physicochemical and functional properties of HPI. In particular, the longest distance between the HPH + HIU_2min_ treatment and the untreated treatment in the first quadrant confirmed that the HPH + HIU_2min_ treatment exerted the most positive influence on the physicochemical and functional properties of HPI.

## 4 Conclusion

This study was the first to investigate the combination of HPH and HIU on the physicochemical and functional properties of HPI. The results of particle size, SEM and AFM indicated that the single process of HPH and HIU could dissociate the larger protein aggregates and facilitate the unfolding of the HPI structure, as validated by the increase of FI and H_0_. The two-stage modification process involved HPH coupled with 2 min of HIU treatment (HPH + HIU_2min_) has proven to amplify this effect, thus further reducing the particle size of HPI, breaking the protein's intermolecular hydrogen bonds and improving its solubility to the highest level. These changes in the physicochemical properties have enhanced HPI functional properties, including WAC/OAC, foaming properties (FC and FS), and emulsifying properties (EAI and ESI). On the other hand, different combinations of HPH + HIU treatments could induce different states of HPI structural changes. As proven in the current study, a longer ultrasonic time of 5 min combined with the HPH could expose more active groups for molecular interaction and cause reaggregation of protein. Further extension of ultrasonic time to 10 min with the HPH treatment could lead to partial disintegration of these protein aggregates and promote HPI unfolding to a certain extent. In summary, this study revealed that the combined HPH with short-time HIU treatment was an efficient method to improve the solubility and functional properties of HPI, facilitating its application in the food industry.

## CRediT authorship contribution statement

**Ruyu Zhang:** Writing – review & editing, Writing – original draft, Methodology, Investigation, Formal analysis, Data curation, Conceptualization. **Wangang Zhang:** Writing – review & editing. **Xuan Dong:** Investigation. **Meng Wai Woo:** Resources. **Siew Young Quek:** Writing – review & editing, Supervision, Resources, Methodology, Funding acquisition.

## Declaration of competing interest

The authors declare that they have no known competing financial interests or personal relationships that could have appeared to influence the work reported in this paper.
